# Study protocol for a pilot randomized crossover trial: Comparing the clinical utility, feasibility, and patient outcomes of remote 3D-printed orthosis fabrication using an innovative heat-reshapable PCL

**DOI:** 10.1177/17589983261430907

**Published:** 2026-03-19

**Authors:** Maryam Farzad, Joy MacDermid, Louis Ferreira, Steven Cuypers

**Affiliations:** 1 Hand and Upper Limb Center, St. Joseph’s Health Center, London, ON, Canada; 2 School of Physical Therapy, Department of Health and Rehabilitation Sciences, Western University, London, ON, Canada; 3 Department of Occupational Therapy, University of Social Welfare and Rehabilitation Sciences, Tehran, Iran; 4 Physical Therapy and Surgery, Western University, London, ON, Canada; 5 Rehabilitation Science, McMaster University, Hamilton, ON, Canada; 6 Department of Mechanical and Materials Engineering/Department of Surgery, Western University, London, ON, Canada; 7 Orfit Industries, Wijnegem, Belgium

**Keywords:** orthosis, 3D print, RCT, CMC OA

## Abstract

**Introduction:**

Orthotic fabrication is a widely used option of the conservative management of carpometacarpal (CMC) osteoarthritis, where conventional low-temperature thermoplastic orthosis requires in-person expertise and adjustments, which limit access to care. Advances in 3D printing and smartphone-based scanning offer opportunities for remote orthosis fabrication. However, the feasibility, accuracy, and patient outcomes compared with traditional methods remain unclear.

**Methods:**

This randomized crossover pilot trial will compare remote 3D-printed orthoses, fabricated using a novel heat re-shapable polycaprolactone (PCL), with conventionally fabricated thermoplastic orthoses in 40 hands with early-stage CMC osteoarthritis. Each participant will receive both interventions in a random order, separated by a 1-week washout period. Outcomes include pain (measured using the Numerical Rating Scale), function (assessed using the QuickDASH), adherence, satisfaction, and clinical utility (evaluated using the QUEST and qualitative interviews). Technical parameters (fabrication time, material properties, weight, and need for adjustments) and the feasibility of smartphone versus high-precision photogrammetry scanning will also be evaluated.

**Results:**

As a pilot study, outcomes will focus on feasibility benchmarks (recruitment, retention, adherence) and variability estimates to inform sample size calculations for a definitive trial. Preliminary comparisons of pain, function, and satisfaction between orthosis types, as well as the usability of smartphone scanning and the adaptability of heat re-shapable PCL, will be reported.

**Discussion/Conclusion:**

This study will provide the first clinical evidence on the feasibility and patient-centred utility of remote orthosis fabrication using an innovative heat re-shapable 3D printing material. Findings will inform whether digital workflows can extend access to hand therapy and support larger-scale trials to evaluate clinical effectiveness and long-term outcomes.

## Key messages

• Remote orthosis fabrication using smartphone scanning and 3D printing will evaluate feasibility and may expand access to conservative care for CMC osteoarthritis.
• Patient-centred factors such as comfort, satisfaction, and adherence are likely to be as important as biomechanical outcomes when evaluating new orthosis fabrication technologies.
• This pilot study provides feasibility data and variability estimates to guide the design of a definitive trial on remote 3D-printed orthoses in clinical practice.

## Introduction

Thumb carpometacarpal (CMC) osteoarthritis is a common degenerative condition leading to pain, weakness, and reduced dexterity, often limiting engagement in daily and occupational tasks. Orthotic intervention remains one of the most frequently recommended conservative treatments, aiming to relieve pain, support joint alignment, and maintain functional use of the hand.^1,2^ However, systematic reviews have indicated that the quality of evidence supporting the effectiveness of orthoses remains low to moderate, with only modest improvements in pain and function observed among selected patients over the medium term.^1,3^ Recent evidence-based guidelines also emphasize that orthoses should not be used in isolation but as part of a comprehensive self-management approach including education, activity modification, and exercise.^4,5^

Low-temperature thermoplastic (LTTP) materials are widely used in hand orthosis fabrication, requiring skilled clinicians to mold, contour, and refine each device to achieve an optimal fit, comfort, and therapeutic outcome. Although this approach remains the clinical standard, it is highly dependent on the therapist’s expertise, often necessitates multiple in-person sessions for fitting and adjustments, and may present challenges related to material durability, patient adherence, and overall satisfaction.^6–8^ Commercial off-the-shelf orthoses, while more accessible, often lack the individualized customization required for effective joint stabilization and long-term comfort.^6^

Advancements in three-dimensional (3D) scanning and printing technologies have introduced new opportunities for personalized orthotic fabrication, enabling high precision, reproducibility, and the potential for remote service delivery.^9–11^ These technologies address the ongoing need for fabrication methods that strike a balance between accessibility, precision, and patient-specific customization. 3D printing, in particular, has emerged as a promising alternative to conventional thermoplastic fabrication, offering advantages such as improved fit, reduced weight, enhanced ventilation, and greater aesthetic adaptability.^9,10^ Comparative studies have demonstrated higher patient satisfaction, better hygiene, and reduced material waste with 3D-printed orthoses compared with thermoplastic orthoses.^10,12–14^ Digital modelling also allows optimization of structural reinforcement and material distribution—capabilities not achievable with conventional LTTP methods.^15^

Despite these advantages, clinical adoption of 3D printing remains limited by material performance constraints, post-processing requirements, and the need for advanced computer-aided design (CAD) and printing expertise.^16^ At the same time, access to specialized hand therapy services remains uneven, particularly for individuals living in remote or underserved areas.^17^ These ongoing challenges have driven interest in remote orthotic fabrication, a digital workflow that integrates 3D hand scanning, cloud-based design, and distributed 3D printing.^18^ This approach enables orthoses to be designed and manufactured without requiring patients to attend in-person fabrication sessions, thereby expanding access to personalized care while maintaining precision and quality control.^19^

However, reducing dependence on in-person evaluations remains challenging due to the complex geometry of the hand, the limited availability and high cost of high-resolution scanners, and the technical expertise required for accurate digital modelling.^20–22^ Although high-precision 3D scanners can produce detailed anatomical data, they are impractical for remote use due to their clinic-based assessment and specialized equipment requirements.^23–25^ Recent advances in smartphone-based 3D scanning technologies, such as photogrammetry and LiDAR-enabled applications, offer a more accessible alternative, enabling clinicians and patients to capture hand geometry remotely with clinically acceptable accuracy.^26–29^

The ubiquity of smartphones and the rapid development of scanning applications have positioned them as practical and low-cost tools for remote clinical imaging across several healthcare domains, including dentistry,^30,31^ making prosthetic sockets^32^ and evaluating keloid scars.^33–35^ While these emerging technologies are promising, they require further validation in clinical settings to determine their accuracy, reliability, and feasibility for routine practice. Further validation is also required to confirm their reliability and feasibility for orthotic design and fabrication workflows. Within this emerging digital model, material selection remains a crucial determinant of clinical usability and patient outcomes. Polycaprolactone (PCL), a biocompatible, low-melting temperature thermoplastic, offers a distinct advantage in that it can be heat reshaped after printing, allowing for post-fabrication adjustment by clinicians or under guided remote supervision^33^ This property addresses a major limitation of commonly used 3D-printed materials such as polylactic acid (PLA) and polyethylene terephthalate glycol (PETG), which cannot be reheated to accommodate changes in fit during rehabilitation.^36,37^ Integrating heat-shapable PCL into a remote 3D fabrication workflow combines the precision of digital modelling with the adaptability of traditional thermoplastics, potentially enhancing fit, comfort, workflow efficiency, and adherence.

Therefore, this pilot randomized crossover study was designed to evaluate the feasibility, clinical utility, and patient acceptability of remote orthotic fabrication using a heat re-shapeable PCL material compared with conventional thermoplastic fabrication in individuals with thumb CMC osteoarthritis. By allowing each participant to experience both fabrication methods, the study will provide within-subject comparisons of comfort, adherence, and satisfaction, while also exploring the accuracy and practicality of smartphone-based scanning technologies for remote orthosis design.

## Methods and analysis

### Study design

This study will employ a randomized crossover design to evaluate the clinical utility, feasibility, and patient outcomes of remote 3D-printed orthosis fabrication using an innovative heat re-shapable polycaprolactone (PCL) filament (SHAP3D, Orfit Industries, Belgium) compared with traditional in-person thermoplastic orthosis fabrication in individuals with CMC osteoarthritis (OA). The primary aim will be to assess patient adherence, functional outcomes, and satisfaction. A secondary aim will be to examine the feasibility and accuracy of smartphone-based 3D scanning compared with high-precision photogrammetry for digital orthotic design.

A within-subjects design will be implemented, allowing each participant to receive both interventions in a randomized sequence for direct comparison within the same individual. Participants will be randomly assigned to one of two sequences: starting with either the conventional thermoplastic orthosis or the remotely fabricated 3D-printed orthosis, followed by crossover to the alternate method after a 1-week washout period to minimize carryover effects.

Participants will be evaluated at multiple time points: baseline (T1), after the first orthosis phase (T2), after the washout period (T3), and after completing the second orthosis phase (T4). All study procedures will adhere to the CONSORT (Consolidated Standards of Reporting Trials) extension for randomized crossover trials. The protocol has been prospectively registered at https://ClinicalTrials.gov (NCT05896410).

### Study setting

This study will be conducted at a university-affiliated hand therapy and research centre. The site provides assessment and treatment for people with upper limb conditions and has facilities for hand function evaluation, 3D scanning, and 3D printing.

### Participants

A convenience sample of 40 hands with CMC osteoarthritis will be recruited, consistent with recommendations for pilot feasibility studies to estimate variability and inform future large-scale trials.

Eligible participants will be 18 years or older, have a clinical diagnosis of CMC OA (Eaton-Littler stage I or II),^38,39^ and have a clinical indication for orthotic immobilization as part of conservative management. All participants must be able to read and communicate in English and be willing to undergo both intervention conditions.

Exclusion criteria will include active skin lesions or dermatological conditions affecting the hand, neurological disorders that alter pain perception or sensory feedback, and prior experience with 3D-printed orthoses for CMC OA. These criteria will ensure a homogeneous sample and reduce confounding related to prior exposure or impaired sensory function.

#### Recruitment

Participants will be identified from the patient population attending hand therapy or surgical consultations for CMC OA at a specialty hand and upper limb care centre. Those meeting eligibility criteria will be invited to participate and provided with written and verbal information about the study. Informed consent will be obtained before enrollment.

Following baseline assessment, participants will be randomly allocated in a 1:1 ratio to one of the two intervention sequences using a computer-generated randomization schedule. The randomization process will be conducted by a researcher not involved in data analysis. Due to the nature of the interventions, blinding of participants and therapists will not be feasible; however, data analysis will be performed by assessors who are blinded to group allocation to minimize bias.

### Intervention

All participants will receive identical standardized education on joint protection, activity modification, and a simple home exercise program. Each participant will also be fitted with two orthoses: one fabricated from low-temperature thermoplastic material (Orfit Colors, Orfit Industries, Belgium) and one produced through 3D printing using a heat re-shapable filament (SHAP3D, Orfit Industries, Belgium). The sequence of interventions will be randomized, and a 1-week washout period will separate the two phases to minimize potential carryover effects.

The conventional orthosis will be fabricated in person by an experienced hand therapist using Orfit Color low temperature thermoplastic material (Orfit Industries, Belgium). A ‘whale-style'^40^ thumb CMC orthosis will be used for all participants, providing radial and palmar stabilization of the first CMC joint while allowing free motion at the metacarpophalangeal and interphalangeal joints. The orthosis will be molded directly on each participant’s hand in a functional position, maintaining thumb opposition and palmar abduction. During fabrication, the therapist will refine contours and edges to optimize comfort, ensure adequate joint stabilization, and minimize pressure points or skin irritation. Each orthosis will be finished with smooth edges, and participants will receive standardized instructions on donning, doffing, hygiene, and recommended wearing schedules. Fabrication time and orthosis weight will be recorded for comparison with the 3D-printed device.

For the remote fabrication, the same whale-style pattern will be replicated digitally to ensure direct comparability between the two fabrication methods. Participants’ hand geometry will be captured using a smartphone-based 3D scanning application based on photogrammetry principles. A high-precision photogrammetry scanner will also be used as a reference standard to assess the accuracy of mobile scanning. The resulting digital models will be uploaded to a secure cloud-based computer-aided design (CAD) platform, where orthoses will be virtually modelled according to a standardized design protocol. The virtual design process will include alignment with anatomical landmarks, the addition of ventilation perforations, and refinement of edges to enhance comfort and stability prior to printing.

Each orthosis will be 3D-printed using SHAP3D (Orfit Industries, Belgium), a newly developed heat shapable filament composed of polycaprolactone. This material allows the orthosis to be reheated and recontoured after printing, combining the precision of additive manufacturing with the adaptability of traditional thermoplastics. Minor post-printing refinements will be performed by locally reheating specific regions to achieve optimal fit and comfort. All printed orthoses will be inspected for dimensional accuracy, surface quality, and smoothness prior to delivery. Standardized verbal and written instructions on orthosis care, application, and precautions will be provided, and participants will also receive a short instructional video to support home use and self-management.

**Figure 1.**
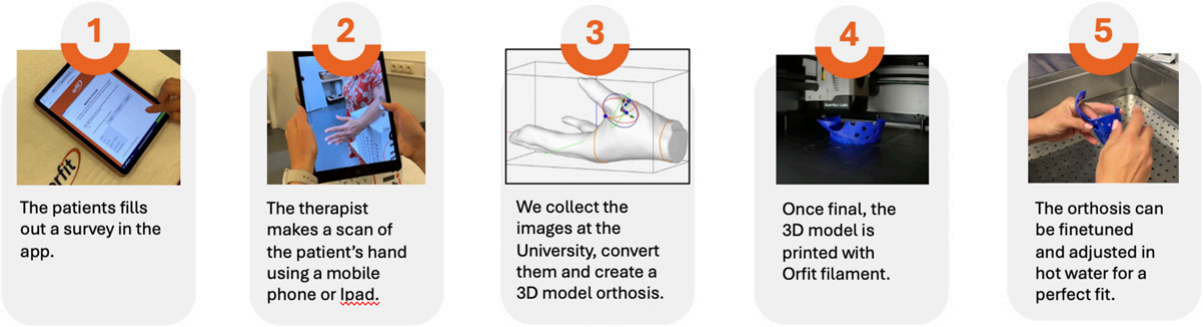
Overview of the remote 3D orthosis fabrication workflow. The process includes five stages: (1) the patient completes an online survey through the app; (2) the therapist scans the patient’s hand using a smartphone or tablet; (3) the images are processed and converted into a 3D model orthosis at the university design platform; (4) the finalized model is printed using Orfit’s SHAP3D heat-reshapable filament; and (5) the orthosis is fine-tuned and adjusted in hot water to ensure a precise fit.

A schematic overview of both fabrication workflows is presented in Figure 1, illustrating the sequential stages of scanning, digital modelling, 3D printing, and post-processing for the remote workflow, alongside the conventional thermoplastic fabrication process.

### Measures

Participants will use each orthosis for a period of 3 weeks, separated by a 1-week washout phase before crossing over to the alternate orthosis. Measurements will be conducted before and after each orthosis phase to evaluate the effects of each intervention on pain, function, and user experience.

### Primary outcomes

Pain will be measured using the Numerical Rating Scale (NRS), a unidimensional, reliable, and sensitive measure of pain widely used in musculoskeletal and degenerative conditions.^41^

#### Functional status

Upper limb function will be assessed using the Quick Disabilities of the Arm, Shoulder and Hand (QuickDASH) questionnaire.^42^ The QuickDASH consists of 11 items, each scored from 0 to 100, with higher scores indicating greater disability.

#### Effectiveness

Effectiveness will be evaluated by comparing the results of pain and function with the orthosis at baseline and after each orthosis using the QuickDASH and NRS. Patients will be asked to monitor their pain intensity during activities and compliance with their orthosis.

### Secondary outcomes

#### Clinical utility

Clinical utility will be explored through a semi-structured interview conducted after participants complete both orthosis trials. The interview will address perceived comfort, fit, usability, and overall satisfaction, as well as practical aspects such as convenience and acceptability of the fabrication method.

Satisfaction will be further assessed using the Quebec User Evaluation of Satisfaction with Assistive Technology (QUEST 2.0), which measures satisfaction with assistive devices across eight domains on a five-point Likert scale.^43^

Complications and adherence will be monitored using a daily diary, in which participants will record wear time, any episodes of discomfort, skin irritation, or device breakage.^44^ Adherence will be categorized as *compliant*, *partially compliant*, or *non-compliant* based on self-reported use (Table 1).

**Table 1.** The quest interview questions.

Features	Summary of questions
Dimension	The size of this orthosis suits my hand.
Weight	This orthosis is lightweight; I can hardly feel the weight.
Adjustment	This orthosis is flexible and easy to use. It is convenient and adjustment is unnecessary.
Safety	My hand is well supported by this orthosis, and my thumb joints are fixed by it. I feel quite secure.
Durability	This orthosis is constructed in an all-in-one design. I feel it is durable.
Simplicity	This orthosis is very easy to put on … the therapist showed me once, and I knew how to wear it. Just by looking at this orthosis, I know which side is the upper or lower side
Comfort	I thought wearing this orthosis helped me with stabilizing my hand joints so they would not move, and it felt very comfortable to me. However, I am worried that when this orthosis gets in the water, it will become wet, and it may be very uncomfortable to wear a wet orthosis. I would not appreciate it if I did anything with water, such as cooking, mopping, cleaning, gardening … That’s another reason I worry about its comfort.
Effectiveness	To me, it’s good that wearing this orthosis helped me by stabilizing my thumb joints. Despite the wetting issue, it was realistic for me.

### Exploratory outcomes

The technical aspects of both fabrication methods will be compared, including total preparation and fabrication time, orthosis weight, and the frequency of repeat or failed fabrications. These measures will help determine the relative feasibility and efficiency of each approach (Table 2).

**Table 2.** Overview of measurements at the different assessment moments.

Measure	Conventional orthosis	3D-printed orthosis
Assessment time points	T1: Baseline	T2: Post-orthosis 1 (3 weeks)
Primary outcome		
Pain (NRS)	x	x
Functional status (Quick DASH)	x	x
Secondary outcomes		
Clinical utility (semi-structured interview)		x
Complications (Patient Diaries)	x	x
Patient compliance (diary reporting)	x	x
Satisfaction (5-point Likert scale)		x
Feasibility & satisfaction with assistive tech (QUEST)		x
Practicality		
Production time	Prospectively assessed	Prospectively assessed
Fabrication costs	Administrative records	Prospectively assessed
Number of visits	Administrative records	Prospectively counted
Technical aspects		
Material properties		x
Orthosis weight		x
Repeat/Failed fabrications		x
Additional outcomes		
Demographics	Intake form	x
Clinical data	Intake form	x
Adverse events (AE form)	x	x

### Statistical analysis and data handling

Data will be analyzed using SPSS version 26 (IBM Corp., Armonk, NY, USA). Descriptive statistics (means, standard deviations, medians, and interquartile ranges) will be used to summarize demographic and baseline characteristics.

Given the randomized crossover design, data will first be examined for potential carryover and period effects. Carryover effects will be evaluated by comparing baseline values of the second intervention phase between groups. If no significant carryover is detected, within-subject comparisons between the two orthosis types will be performed using the paired t-test or the Wilcoxon signed-rank test, as applicable, for non-normally distributed data. The Shapiro–Wilk test will be applied to assess the normality of continuous variables.

The primary outcomes pain intensity (NRS) and upper limb function (QuickDASH)—will be analyzed using within-subject comparisons to evaluate differences between the 3D-printed and conventional orthoses. Secondary outcomes, including clinical utility, satisfaction, and fabrication characteristics (time, weight, and re-fabrication rate), will be analyzed descriptively and compared using the same approach where applicable. The significance level will be set at *p* < 0.05.

Qualitative data from semi-structured interviews will be analyzed using conventional content analysis to identify recurring themes related to comfort, practicality, and perceived usefulness of each fabrication method. Two independent reviewers will code and categorize responses to enhance reliability and accuracy.

As this is a pilot feasibility study, the analysis will focus on estimating effect sizes, feasibility indicators, and confidence intervals rather than hypothesis testing. Findings will inform refinements to study design, procedures, and sample size calculations for a future definitive trial.

## Discussion

This protocol outlines a randomized crossover pilot trial designed to evaluate the feasibility and clinical utility of a remote 3D orthotic fabrication workflow compared with conventional thermoplastic splinting for individuals with thumb carpometacarpal osteoarthritis. The newly developed workflow^19^ also introduces the use of SHAP3D, a heat re-shapable polycaprolactone filament combining digital precision with the adjustability of traditional thermoplastic materials.

Three-dimensional scanning and printing technologies have been increasingly explored in hand therapy^45,46^; however, evidence on their practical feasibility in remote fabrication remains limited. This study aims to address that gap by examining whether smartphone-based 3D scanning and remote computer-aided design can produce orthoses that are comparable in comfort, functionality, and patient experience to those fabricated in person. The inclusion of both mobile and high-precision scanning methods presents an opportunity to evaluate the clinical accuracy and usability of readily available digital tools, thereby supporting future applications in telehealth and remote care.

The crossover design allows each participant to act as their own control, strengthening internal validity by minimizing individual variability. However, potential carryover effects remain a consideration despite the inclusion of a washout period. The use of validated outcome measures for pain and function ensures consistent assessment across intervention phases, although the limited sample size precludes firm conclusions about clinical effectiveness.

While this study introduces an innovative heat re-shapable filament, a standardized protocol for reheating and reshaping has yet to be developed. This may lead to variability in how adjustments are made, which future studies should address through protocol refinement and mechanical testing. As with all pilot studies, the small sample and potential selection bias limit the generalizability of findings, emphasizing the exploratory nature of this work.

Despite these limitations, the study provides an important step toward understanding the feasibility and acceptability of remote orthotic fabrication. The results will help refine workflow procedures, assess scanning reliability, and evaluate material usability in preparation for larger-scale clinical studies. This work contributes to the growing effort to make orthotic care more accessible through digital and remote methods.

## Conclusion

This pilot study aims to generate preliminary evidence on the feasibility, clinical utility, and patient experience of remote 3D-printed orthoses made with a heat re-shapable filament, compared to traditional thermoplastic splints. The findings will inform future research focused on optimizing digital design workflows, developing standardized reshaping procedures, and evaluating long-term clinical outcomes. While the results will not determine efficacy or signal a change in clinical practice, they are expected to provide valuable feasibility data and methodological guidance for future large-scale investigations.
